# Elucidation of Critical Epitope of Anti-Rat Podoplanin Monoclonal Antibody PMab-2

**DOI:** 10.1089/mab.2018.0025

**Published:** 2018-08-01

**Authors:** Yoshikazu Furusawa, Shinji Yamada, Shunsuke Itai, Takuro Nakamura, Masato Fukui, Hiroyuki Harada, Mika K. Kaneko, Yukinari Kato

**Affiliations:** ^1^ Department of Antibody Drug Development, Tohoku University Graduate School of Medicine, Sendai, Japan.; ^2^ New Industry Creation Hatchery Center, Tohoku University, Sendai, Japan.; ^3^ ZENOAQ RESOURCE CO., LTD., Koriyama, Japan.; ^4^ Department of Oral and Maxillofacial Surgery, Graduate School of Medical and Dental Sciences, Tokyo Medical and Dental University, Tokyo, Japan.

**Keywords:** podoplanin, PDPN, PMab-2, epitope mapping

## Abstract

Rat podoplanin (rPDPN) is a recognized lymphatic endothelial cell marker and is expressed on the podocytes of kidney and type I lung alveolar cells. rPDPN is a type I transmembrane sialoglycoprotein and induces platelet aggregation via the C-type lectin-like receptor-2 of platelets. It comprises four platelet aggregation-stimulating (PLAG) domains: PLAG1–3, present in the N-terminus, and PLAG4, in the center of the PDPN protein. Previously, we developed a mouse anti-rPDPN monoclonal antibody clone, PMab-2, by immunizing the PLAG2 and PLAG3 domains of rPDPN. PMab-2 has applications in Western blot, flow cytometry, and immunohistochemical analyses for detection of both normal and cancer cells. However, the binding epitope of PMab-2 remains to be determined. Herein, we investigated the epitope of PMab-2 using enzyme-linked immunosorbent assay, immunohistochemical analysis, and flow cytometry. The results revealed that the critical epitope of PMab-2 is Leu46 and Glu47 of rPDPN.

## Introduction

Rat podoplanin (rPDPN) is a recognized lymphatic endothelial cell marker and is expressed on the podocytes of kidney and type I lung alveolar cells.^(1–4)^ rPDPN is a type I transmembrane sialoglycoprotein and induces platelet aggregation via the C-type lectin-like receptor-2 (CLEC-2) of platelets.^(5–7)^ It comprises four platelet aggregation-stimulating (PLAG) domains: PLAG1–3, present in the N-terminus,^(1)^ and PLAG4, in the center of the rPDPN protein.^(8)^

PMab-2 monoclonal antibody (mAb) was previously produced against PLAG domain of rPDPN^(9)^; therefore, PMab-2 could neutralize interactions between rPDPN and CLEC-2.^(5–7)^ PMab-2 is applicable in Western blot, flow cytometry, and immunohistochemical analyses.

In this study, we determined the binding epitope of PMab-2 to rPDPN using flow cytometry and enzyme-linked immunosorbent assay (ELISA).

## Materials and Methods

### Cell line

Chinese hamster ovary (CHO)-K1 was purchased from the American Type Culture Collection (ATCC, Manassas, VA). CHO/rPDPN was produced in our previous study.^(9)^ The rPDPN mutation plasmids were transfected into CHO-K1 cells using Lipofectamine LTX (Thermo Fisher Scientific, Inc., Waltham, MA). CHO/rPDPN and transiently transfected cells were cultured in RPMI 1640 medium (Nacalai Tesque, Inc., Kyoto, Japan), supplemented with 10% heat-inactivated fetal bovine serum (Thermo Fisher Scientific, Inc.), 100 U/mL of penicillin, 100 μg/mL of streptomycin, and 25 μg/mL of amphotericin B (Nacalai Tesque, Inc.) at 37°C in a humidified atmosphere of 5% carbon dioxide and 95% air.

### Production of rPDPN point mutants

The complementary DNA of rPDPN was subcloned into a pcDNA3 vector (Thermo Fisher Scientific, Inc.). Substitutions of amino acids to alanine in rPDPN sequence were performed by the QuikChange Lightning Site-Directed Mutagenesis Kit (Agilent Technologies, Inc., Santa Clara, CA).

### Flow cytometry

Cells were harvested after brief exposure to 0.25% trypsin/1 mM ethylenediaminetetraacetic acid (Nacalai Tesque, Inc.). After washing with 0.1% bovine serum albumin in phosphate-buffered saline (PBS), the cells were treated with PMab-2 for 30 minutes at 4°C, followed by treatment with Alexa Fluor 488-conjugated anti-mouse IgG (1:1000; Cell Signaling Technology, Inc., Danvers, MA). Fluorescence data were acquired using the Cell Analyzer EC800 (Sony Corp., Tokyo, Japan).

### Enzyme-linked immunosorbent assay

Synthesized rPDPN peptides using PEPscreen (Sigma-Aldrich Corp., St. Louis, MO) were immobilized on Nunc MaxiSorp 96-well immunoplates (Thermo Fisher Scientific, Inc.) at 10 μg/mL for 30 minutes at 37°C. After blocking with Superblock T20 (PBS) blocking buffer (Thermo Fisher Scientific, Inc.), the plates were incubated with purified PMab-2 (10 μg/mL), followed by a 1:2000 dilution of peroxidase-conjugated anti-mouse IgG (Agilent Technologies, Inc.). The enzymatic reaction was conducted using 1-Step Ultra TMB-ELISA (Thermo Fisher Scientific, Inc.). Optical density was measured at 655 nm using an iMark Microplate Reader (Bio-Rad Laboratories, Inc., Berkeley, CA). These reactions were performed at 37°C with a total sample volume of 50–100 μL.

### Immunohistochemical analyses

Histological sections (4 μm thick) of rat tissues were directly autoclaved in citrate buffer (pH 6.0; Nichirei Biosciences, Inc., Tokyo, Japan) for 20 minutes. After blocking with Superblock T20 (PBS) blocking buffer, sections were incubated with PMab-2 (1 μg/mL) or PMab-2 (1 μg/mL) plus peptides (5 μg/mL) for 1 hour at room temperature, treated using an EnVision+ Kit (Agilent Technologies, Inc.) for 30 minutes. Color was developed using 3,3-diaminobenzidine tetrahydrochloride (Agilent Technologies, Inc.) for 2 minutes and counterstained with hematoxylin (FUJIFILM Wako Pure Chemical Corporation, Osaka, Japan).

## Results and Discussion

We previously developed a mouse anti-rPDPN mAb, PMab-2, by immunizing the PLAG domain of rPDPN.^(9)^ In this study, we produced point mutants of rPDPN using recombinant proteins and synthesized peptides and investigated the epitope of PMab-2 critical for rPDPN detection.

**FIG. 1.**
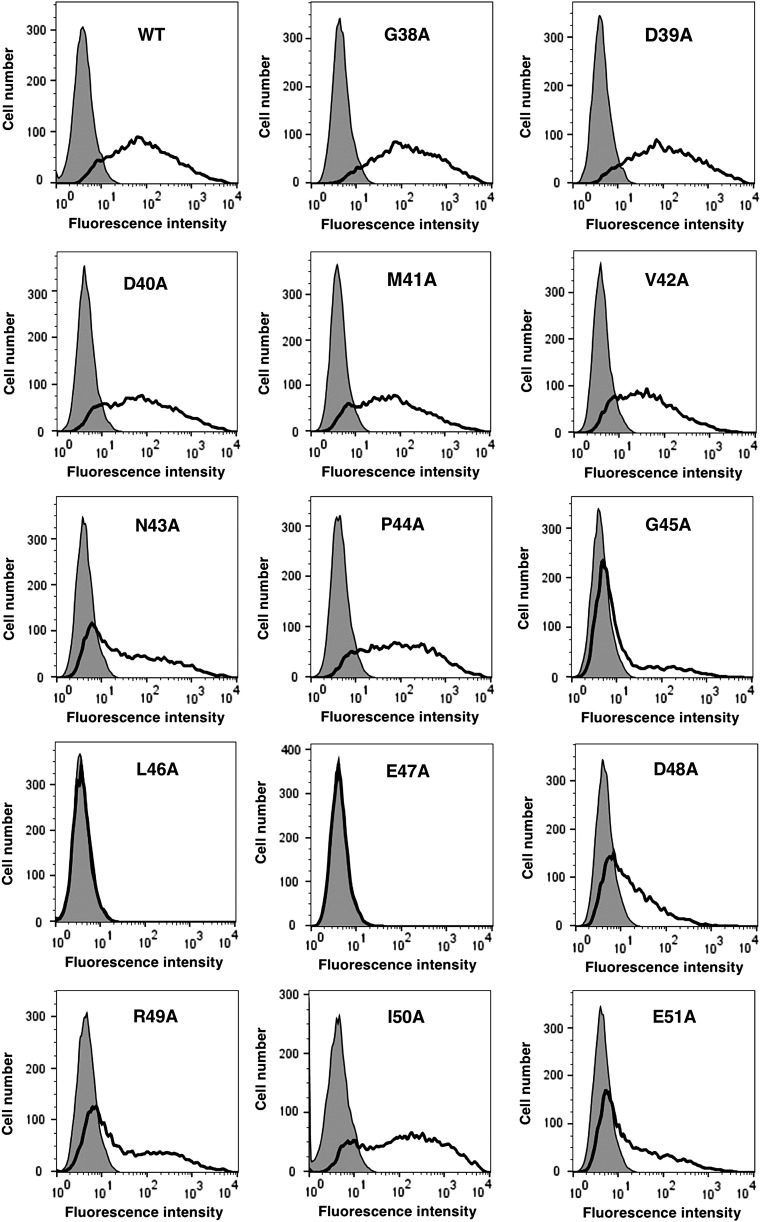
Epitope mapping of PMab-2 using point mutants of rPDPN. Point mutants of rPDPN were analyzed using flow cytometry. Point mutants were expressed on CHO-K1 cells and were then incubated with PMab-2 (2 μg/mL) or buffer control for 30 minutes at 4°C, followed by treatment with corresponding secondary antibodies.

We produced a series of point mutants of rPDPN using a QuikChange Lightning Site-Directed Mutagenesis Kit because PMab-2 was produced by immunizing mice with amino acids 38–51 of rPDPN. Using flow cytometry, we found that PMab-2 recognized G38A, D39A, D40A, M41A, V42A N43A, P44A, G45A, D48A, R49A, I50A, and E51A (Fig. 1). However, it did not recognize L46A and E47A, thus indicating that Leu46 and Glu47 of rPDPN are crucial for PMab-2 detection.

Next, we synthesized a series of peptides: point mutants of 38–51 amino acids of rPDPN (Table 1). Using ELISA, we found that PMab-2 detected G38A, D39A, D40A, M41A, V42A N43A, P44A, G45A, D48A, R49A, I50A, and E51A; in contrast, it did not recognize L46A and E47A, thus confirming the result obtained via flow cytometry.

**Table 1.** Determination of PMab-2 Epitope by Enzyme-Linked Immunosorbent Assay

*Mutation*	*Sequence*	*PMab-2*
G38A	ADDMVNPGLEDRIE	+++
D39A	GADMVNPGLEDRIE	+++
D40A	GDAMVNPGLEDRIE	+++
M41A	GDDAVNPGLEDRIE	+++
V42A	GDDMANPGLEDRIE	+++
N43A	GDDMVAPGLEDRIE	+
P44A	GDDMVNAGLEDRIE	+++
G45A	GDDMVNPALEDRIE	+
L46A	GDDMVNPGAEDRIE	−
E47A	GDDMVNPGLADRIE	−
D48A	GDDMVNPGLEARIE	+++
R49A	GDDMVNPGLEDAIE	+++
I50A	GDDMVNPGLEDRAE	+++
E51A	GDDMVNPGLEDRIA	+++

+++, OD655≧1.0; +, 0.1≦OD655<0.6; −, OD655<0.1.

**FIG. 2.**
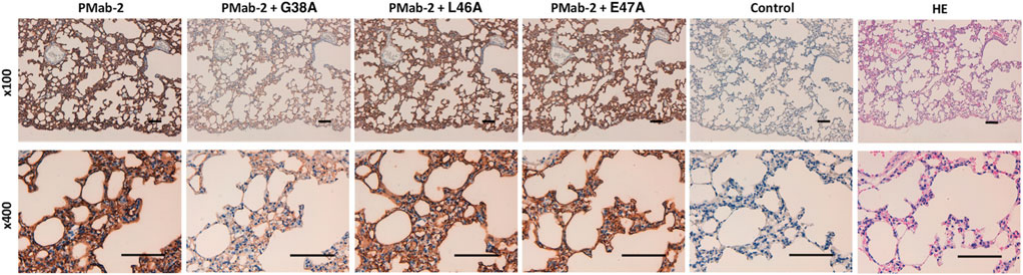
Immunohistochemical analyses using rat lung tissues. Histological sections of lung were directly autoclaved in citrate buffer for 20 minutes. After blocking with blocking buffer, sections were incubated with PMab-2 (1 μg/mL) or PMab-2 (1 μg/mL) plus peptides (5 μg/mL), followed by an EnVision+ Kit. Scale bar = 100 μm. HE, hematoxylin and eosin.

**FIG. 3.**
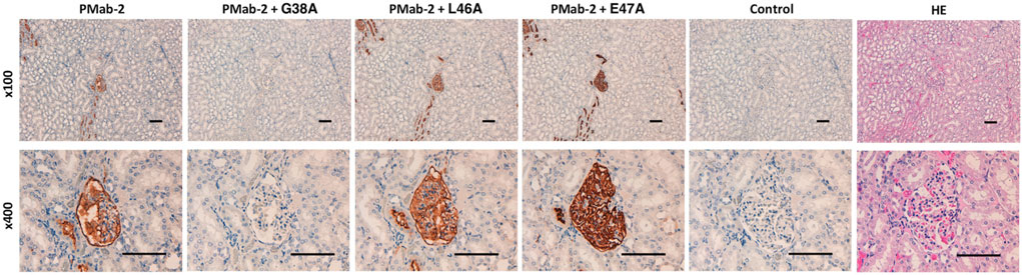
Immunohistochemical analyses using rat kidney tissues. Histological sections of kidney were directly autoclaved in citrate buffer for 20 minutes. After blocking with blocking buffer, sections were incubated with PMab-2 (1 μg/mL) or PMab-2 (1 μg/mL) plus peptides (5 μg/mL), followed by an EnVision+ Kit. Scale bar = 100 μm.

**FIG. 4.**
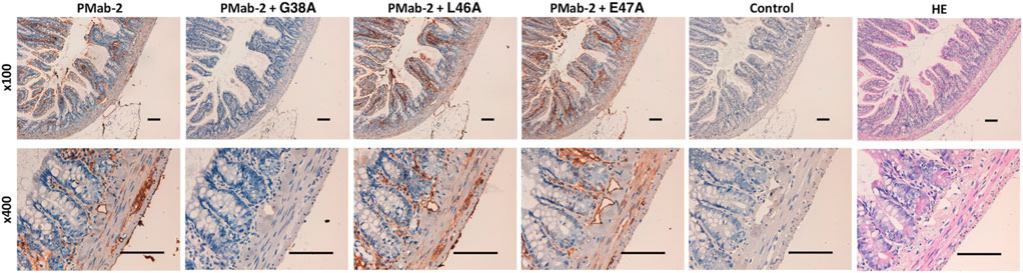
Immunohistochemical analyses using rat colon tissues. Histological sections of colon were directly autoclaved in citrate buffer for 20 minutes. After blocking with blocking buffer, sections were incubated with PMab-2 (1 μg/mL) or PMab-2 (1 μg/mL) plus peptides (5 μg/mL), followed by an EnVision+ Kit. Scale bar = 100 μm.

We performed a blocking assay using immunohistochemistry. PMab-2 reacted with type I alveolar cells (Fig. 2), renal podocytes (Fig. 3), and lymphatic endothelial cells of colon (Fig. 4). These reactions were completely or partially neutralized by G38A; in contrast, L46A and E47A did not block the reactions of PMab-2 with rat tissues, thus indicating that L46A and E47A of rPDPN are critical for PMab-2 detection.

**FIG. 5.**
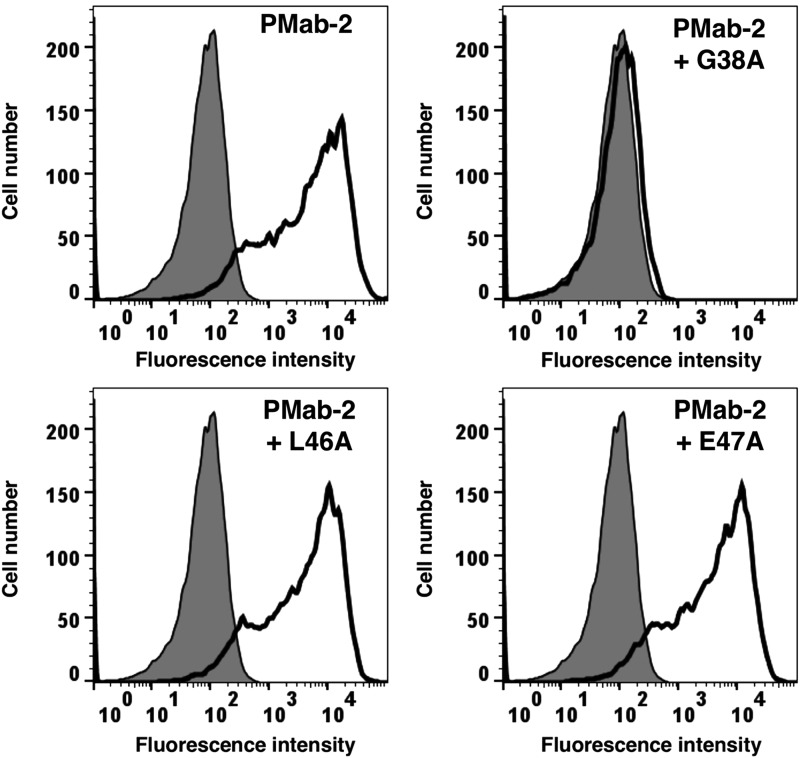
Flow cytometry using PMab-2 and point mutants of rPDPN. PMab-2 (1 μg/mL) or PMab-2 (1 μg/mL) + peptides (G38A, L46A, and E47A; 10 μg/mL) were treated with CHO/rPDPN cells for 30 minutes at 4°C, followed by addition of secondary antibodies.

In addition, we performed a blocking assay using flow cytometry and found that PMab-2 reacted with the CHO/rPDPN cell line (Fig. 5). This reaction was completely neutralized by G38A; in contrast, L46A and E47A did not block the reaction of PMab-2 with CHO/rPDPN, which indicated that Leu46 and Glu47 of rPDPN are critical for PMab-2 detection.

**FIG. 6.**
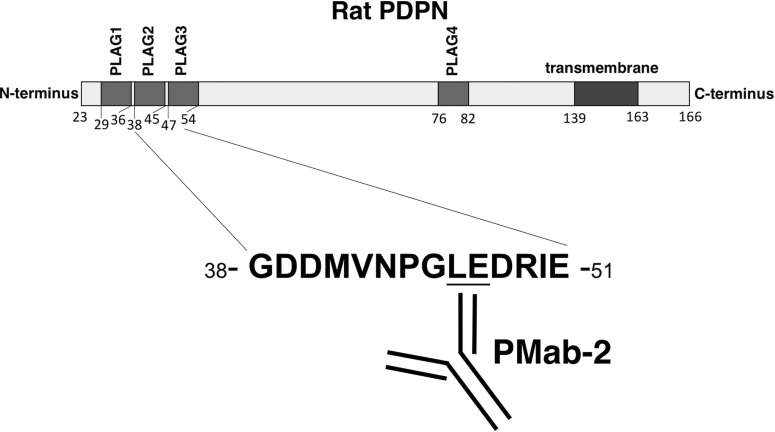
Illustration of rPDPN and epitope of PMab-2. rPDPN possesses four PLAG domains. PMab-2 was established by immunizing PLAG2–3 domains. Leu46 and Glu47 are critical amino acids for PMab-2 recognition to rPDPN. PLAG, platelet aggregation stimulating.

Taken together, the critical epitope of PMab-2 comprises Leu46 and Glu47 of rPDPN (Fig. 6). These findings could be applied to the production of more functional anti-rPDPN mAbs.
